# Prediction of Deleterious Nonsynonymous Single-Nucleotide Polymorphism for Human Diseases

**DOI:** 10.1155/2013/675851

**Published:** 2013-01-30

**Authors:** Jiaxin Wu, Rui Jiang

**Affiliations:** MOE Key Laboratory of Bioinformatics and Bioinformatics Division, TNLIST/Department of Automation, Tsinghua University, Beijing 100084, China

## Abstract

The identification of genetic variants that are responsible for human inherited diseases is a fundamental problem in human and medical genetics. As a typical type of genetic variation, nonsynonymous single-nucleotide polymorphisms (nsSNPs) occurring in protein coding regions may alter the encoded amino acid, potentially affect protein structure and function, and further result in human inherited diseases. Therefore, it is of great importance to develop computational approaches to facilitate the discrimination of deleterious nsSNPs from neutral ones. In this paper, we review databases that collect nsSNPs and summarize computational methods for the identification of deleterious nsSNPs. We classify the existing methods for characterizing nsSNPs into three categories (sequence based, structure based, and annotation based), and we introduce machine learning models for the prediction of deleterious nsSNPs. We further discuss methods for identifying deleterious nsSNPs in noncoding variants and those for dealing with rare variants.

## 1. Introduction

Understanding the relationship between phenotype and genotype is a fundamental problem in genetics. Of particular interest, the identification of genetic risk factors underlying human inherited diseases has long been a goal in human and medical genetics. Since genetic variation is believed to be the major factor that stimulates the diversity between individuals [1], considerable efforts have been taken to understand associations between human genetic variants and their phenotypic effects [2]. A number of successful stories have shown that such efforts are helpful in capturing the causative variants which affect human inherited diseases, providing important information for grasping genetic bases of complex diseases, and further promoting the prevention, diagnosis, and treatment of these diseases [3]. Nevertheless, recent studies have shown that the number of genetic variants is huge, more than 3.5 million variants in the whole genome for a single individual, roughly corresponding to 1,000 variants per megabase pair [4, 5], making the identification of causative variants a task of finding needles in stacks of needles. Furthermore, it has also been shown that although most genetic variants exist common in a population, there also exists a nonnegligible number of variants that occur in very low frequency, making established statistical methods for identifying such rare variants ineffective. Hence, the development of novel computational methods to identify causative variants now receives more and more attentions.

Genetic variants can typically be classified into several categories, including single-nucleotide polymorphisms (SNPs), small insertions and deletions, and structural variants [4]. Among these types of variants, single-nucleotide polymorphisms (SNPs) that occur in single bases of DNA sequences account for a majority of all genetic variants. It has been estimated that there exist nearly 10 million SNPs in the human genome, nearly one SNP for every 290 base-pairs. The vast number of SNPs along with growing functional annotations of the human genome sequence may provide plenty of knowledge to grasp links between genetic and phenotypic variations [6]. Particularly, as an important type of SNP, a nonsynonymous single-nucleotide polymorphism (nsSNP) occurring in a protein coding region alters the encoded amino acid sequence, potentially affects protein structure and function, and further causes human inherited diseases. It has been reported that nsSNPs constitute more than 50% of the mutations known to be involved in human inherited diseases [7] and each person may hold 24,000–40,000 nsSNPs [8]. It is also believed that although most of the susceptible deleterious nsSNPs are related to individual Mendelian diseases, functional changes aroused by nsSNPs will be of importance for complex diseases [8]. Therefore, more effort should be paid for studying the candidate deleterious nsSNPs [9].

The identification of genetic variants that are associated with human diseases is often undertaken using either a family-based linkage analysis or a population-based association study. In a linkage analysis, susceptible disease-causing loci (usually between 1 and 5 million bp in length) are mapped by identifying genetic markers that are coinherited with a query phenotype. Linkage analysis has poor prediction power for difficultly collecting family-based sequence data and poor performance for complex diseases which are caused by the combination of effects of several susceptible genetic variants and their interactions with environmental factors [10]. An association study compares frequencies of occurrence of genetic variants between a case population and a control population to detect associations between genetic variants and phenotypes [11]. With recent advances in high-throughput experimental techniques, association studies are now often conducted in genomewide scale, often referred to as genomewide association (GWA) studies. Although such a GWA study has shown some success in the past few years, it suffers from serious multiple testing problem when applied to a number of markers in a large population, and its basic hypothesis of Common Disease Common Variant (CDCV) has been challenged by the fact that both common variants and rare variants may be involved in the pathogenesis of common diseases.

To overcome these limitations and serve as a complementary category of these traditional statistical methods, computational approaches that rely on properties of variants instead of experimental data of patients have been designed for the detection of deleterious variants, with the growing functional annotations of the human genome sequence. Although such methods may never be accurate enough to replace wet-lab experiments, they may help in identifying and prioritizing a small number of susceptible and tractable candidate nsSNPs from pools of available data [1]. Recent studies [9–21] have shown that computational methods are capable of well estimating the functional effects of nsSNPs. These approaches may take advantage of structure information, sequence information, and annotations as classification features, as well as logistic regression [21], neural networks [1], Bayesian models [5], and other statistical approaches [18] as classifiers. 

In this paper, we first summarize the databases for collecting nsSNP data and provide a framework of nsSNP function prediction methodology. We survey existing deleterious nsSNPs prediction methods and summarize the prediction features conducted in prediction models and the prediction algorithms to distinguish the deleterious nsSNPs. Then, we discuss computational methods that use comparative genomics to predict deleteriousness of nsSNPs in both coding and noncoding regions. We also look at prioritization methods for disease-specific nsSNPs detection and discuss deleterious nsSNPs prediction methods for rare variants detection. Finally, we suggest using multiple prediction algorithms to enhance the prediction power and discuss challenges and likely future improvements of such methods.

## 2. Databases for nsSNPs

Many popular databases present useful information of nsSNPs. Particularly, as shown in Table 1, deleterious nsSNPs are mainly collected in four databases: the Online Mendelian Inheritance in Man (OMIM) database [22], the Human Gene Mutation Database (HGMD) [12], the UniProt/Swiss-Prot database [13], and the Human Genome Variation database (HGVbase) [14]. Other popular databases like the single-nucleotide polymorphism database (dbSNP) [15], the Protein Mutant Database (PMD) [16], and the database for nonsynonymous SNP's function prediction (dbNSFP) [9] are also important for collecting nsSNP data (also shown in Table 1). 

The Online Mendelian Inheritance in Man (OMIM) is a powerful, comprehensive, and widely used database for collecting molecular relations between genetic variations and phenotypes. OMIM contains information of all known Mendelian disorders and their associated genes. Updated to October 23, 2012, OMIM has collected 21,458 entries of possible links between 4,753 phenotypes and over 12,000 genes, and 2,883 genes with phenotype-causing mutations.

The Human Gene Mutation Database (HGMD) records all germ-line disease-causing mutations and deleterious polymorphisms published in the literature. HGMD provides two versions of databases, one is for academic or nonprofit users, and the other is for professional usage. Updated to March 2012, the total mutation data collected in HGMD nonprofit version is 92,715, while the total mutation data in HGMD Professional version is 130,522.

The UniPROT/SWISS-PROT database is a high quality, manually curated, comprehensive protein sequence database, integrating information from the scientific literature and computational analysis. SWISS-PROT provides convincing protein sequences and annotations, such as protein function descriptions and domain structures. Updated to September 2012, UniProtKB/Swiss-Prot contains 538,010 sequence entries and 190,998,508 amino acids abstracted from 213,490 documents, including more than 67,000 nsSNPs.

The Human Genome Variation database (HGVbase) is an accurate, high-quality, and nonredundant database for comprehensive catalog of normal human gene and genome variation, especially SNPs. HGVbase provides both neutral polymorphisms and disease-related mutations. Updated to July 2005 (released 16.0), HGVbase contains 8,924,237 entries, including more than 20,000 coding SNPs and about 11,000 nsSNPs.

The single-nucleotide polymorphism database (dbSNP) is a comprehensive repository for single-nucleotide substitutions, short deletion, and insertion polymorphisms. Data in dbSNP can be combined with other available NCBI genomic data and freely downloaded in a variety of forms. Updated to February 2010, dbSNP has collected over 184 million submissions representing more than 64 million distinct variants for 55 organisms, including more than 70,000 SNPs.

The Protein Mutant Database (PMD) [16] is a literature-based database for protein mutants, providing information of amino acid mutations at specific positions of proteins and the structural alterations. Each entry in the database corresponds to one article which may describe one or several protein mutants. Updated to 26 Mar 2007, PMD collects 45,239 entries and 218,873 mutants, including 54,975 nsSNPs occurring in 4,675 proteins.

The database for nonsynonymous SNPs' functional predictions (dbNSFP) [9] is a newly published database, providing both the information about nsSNPs and prediction scores from four popular algorithms (SIFT [17], PolyPhen-2 [18], LRT [19], and MutationTaster [5]) along with a conservation score (PhyloP) [10]. The dbNSFP is the first known integrated database of functional predictions from multiple algorithms for broad collection of human nsSNPs. Updated to March 27, 2009, dbNSFP includes a total of 75,931,005 entries, which covers 64,646,969 nsSNPs in the human genome.

## 3. Software Tools for Predicting Functional Implication of nsSNPs

With the accelerating advancement of high-throughput experimental techniques, annotations about functional elements in the human genome now become widely available; accordingly a variety of information can be used to study the deleteriousness of an nsSNP. A number of methods have been proposed for the prediction of deleterious nsSNPs, along with friendly web-based interactive software for users to facilitate their own research. In Table 2, we list eleven widely used tools, including SIFT [17], PolyPhen [2], SNAP [1], MSRV [11], LRT [19], PolyPhen-2 [18], MutationTaster [5], KGGSeq [23], SInBaD [21], GERP [24], and PhyloP [10]. The input data for a prediction tool usually requires the protein sequence or protein ID, the amino acid substitution, position of the substitution, chromosome, and/or sequence alignment. After providing all the required input data in the right format, the tools can run automatically and return the predication results, which are usually predictive scores ranging from 0 to 1. 

**Figure 1:**
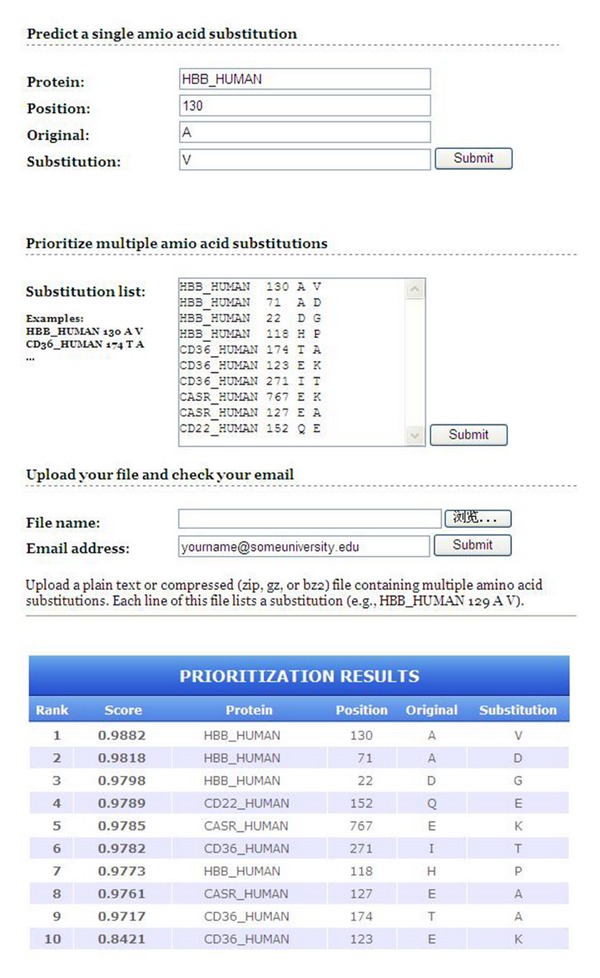
Web interface of MSRV.

Taking MSRV as an example, the input data for predicting a single amino acid substitution that results from a single base alternation in protein coding sequence includes the protein name, the amino acid substitution, and position of the substitution in protein sequence, and the output data includes the prediction score ranging from 0 to 1, where 0 stands for neutral nsSNP and 1 means deleterious nsSNP. For prioritizing multiple amino acid substitutions, users can directly paste their substation lists in the required format to the website or upload their data from local computer. The outputs are the ranking list containing all the attached substitution and their scores (as shown in Figure 1).

**Figure 2:**
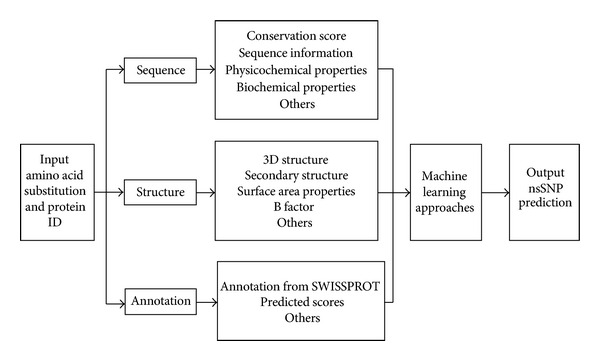
Typical procedure for deleterious nsSNPs detection.

Typically, the deleterious nsSNPs prediction problem is formulated as a binary classification model using diverse genomic data as features to compare the deleterious nsSNP with neutral nsSNP. The typical procedure is shown in Figure 2. Users should provide the information about protein ID or sequence, amino acid substitution, and/or multiple sequence alignment. After inputing all the required information, the classification tools can be implemented by extracting their own features and setting up the new classification model automatically. Finally, the deleterious score or the classification result may output by the tools. Classification features are collected and computed using sequence information, protein structural information, and/or annotations from known databases or prediction results. Sequence-based deleterious nsSNPs prediction methods usually take advantage of biochemical properties, physiochemical properties, sequence information, the evolutionary information of proteins, and the predicted 1D or 2D structure of proteins. Structure-based prediction methods may search a protein structure database and get some structural features for further classification. Annotation-based methods may take annotations from SWISS-PROT database [13] or use some published tools to get the preliminary scores for the query nsSNPs. In the next section, we focus on the eleven computational tools to analyze the deleterious nsSNPs prediction problem from the view of extracted features and classification methods.

## 4. Features for Characterizing nsSNPs 

To fully capture diverse potential properties of deleterious nsSNPs, existing prediction tools take advantage of different types of features including sequencing-based information, structure-based information, and/or annotations to wholesomely carry out the classification of the deleterious nsSNPs from the neutral ones.

### 4.1. Sequencing-Based Information Provides the Strongest Signal for the Prediction Problem

Once a protein sequence containing the query nsSNP is provided, sequence-based deleterious nsSNP prediction methods calculate some specific features according to the sequence of the gene that contains the nsSNP and the location of the nsSNP in the DNA sequence, and/or look up in some databases to collect biochemical properties or physicochemical properties of the nsSNP or resulting single amino acid polymorphism. The most commonly utilized feature based on protein sequence for the query nsSNP is the conservation information calculated in different ways. Usually, people search the protein sequence against a sequence database to find sequences of homologous proteins. A multiple sequence alignment of the homologous sequences reveals what positions have been conserved throughout evolutionary time, and these positions are inferred to be important for function [8]. There are also many other ways to extract the classification features for nsSNPs according to the protein sequence where the nsSNPs locate [5, 25].

#### 4.1.1. Conservation Scores

As an important feature for studying the deleteriousness of an nsSNP, the conservation score is used by most of prediction methods with their own way of calculation. The estimation of the deleteriousness of an nsSNP is based on the fact that sequences observed among living organisms are those that have not been removed by natural selection. In addition, comparative sequence analysis based on phylogenetic information by quantifying evolutionary changes in genes or genomes to find out the conserved positions that have evolved too slowly to be neutral can be identified [4]. Although evolutionary models may not identify all deleterious mutations, they provide a probabilistic framework in which the subset of deleterious mutations that disrupt highly conserved amino acid positions can be accurately identified [19]. 

Genome sequencing of a large number of closely related species makes it possible to develop better parameterized evolutionary models that more accurately predict human deleterious mutations [19]. Therefore, given a protein sequence as input, a sequence database is needed to find homologous sequences for the protein. A multiple sequence alignment of the homologous sequences reveals what positions have been conserved throughout evolutionary time, and these positions are inferred to be important for function [8]. The conservation-based prediction method then scores each nsSNP based on the amino acid appearing in the multiple alignment and the severity of the amino acid change. An amino acid that is not present at the substitution site in the multiple alignment can still be predicted to be neutral if there are amino acids with similar physiochemical properties present in the alignment [8]. 

There are many ways to compute the conservation score for every query nsSNP. PolyPhen identifies homologues of the input sequences via a BLAST [26] search of the NRDB database and uses the new version of the PSIC (position-specific independent counts) software [27] to calculate the profile matrix, whose elements of the matrix (profile scores) are logarithmic ratios of the likelihood of a given amino acid occurring at a particular site to the likelihood of this amino acid occurring at any site (background frequency). PolyPhen computes the absolute value of the difference between profile scores of both allelic variants in the polymorphic position. Besides the PSIC score, PolyPhen-2 also uses the sequence identity to the closest homologue carrying any amino acid that differs from the wild-type allele at the site of the mutation, congruency of the mutant allele to the multiple alignment, and alignment depth (excluding gaps) at the site of the mutation. PhyloP performs an exact *P* value computation under a continuous Markov substitution model to compute the conservation score that measures interspecies conservation at each SNP position. MSRV provides an easy and effective way to calculate the conservation scores for the original and substitute amino acid, which are the frequencies of occurrences of the amino acids in the corresponding position of the Pfam multiple sequence alignment. The same features are also used by the MutationTaster algorithm and the SNAP algorithm. The LRT method utilizes the log likelihood ratio of the conserved relative to neutral model to measure the deleteriousness of an nsSNP, with the null model that each codon is evolving neutrally with no difference in the rate of nonsynonymous to synonymous substitution and the alternative model that the codon has evolved under negative selection with a free parameter for the nonsynonymous to synonymous ratio [19]. 

#### 4.1.2. Sequence Information

Pure sequence information of the protein containing the nsSNP may offer useful indications that helped to identify the deleterious nsSNPs. Different methods adopt different ways to exhibit the usage of protein sequence. PolyPhen uses the characterization of the substitution site as a feature, while PolyPhen-2 employs CpG context of transition mutations. MutationTaster also computes a large number of features to grasp the potential difference between the deleterious nsSNPs and the nondeleterious nsSNPs, one of them is the length of protein, which checks if the resulting protein will be elongated, truncated, or whether nonsense-mediated mRNA decay is likely to occur, another is splice site analysis, which analyzes potential splice site changes.

#### 4.1.3. Physicochemical Properties

It is believed that the physicochemical properties of proteins, especially the changes of physicochemical properties before and after amino acid changes, may present valuable information about how an amino acid substitution may lead to structural or functional changes of a protein. MSRV adopts six physicochemical properties of amino acids, including molecular weight, pI value, hydrophobicity scale, and relative frequencies for the occurrences of amino acids in the secondary structures (helices, strands, and turns) of proteins with known secondary structural information. Six properties are calculated under four situations that are the properties of the original amino acids, properties of the substituted amino acids, properties calculated in a window-sized situation that includes the neighbors of the original amino acids in the query protein sequence, and properties calculated in a column-weighted circumstance in which the query protein sequence is aligned with its homologous proteins. The authors also exploit three more situations which consider the property changes of the substitute amino acid from the original amino acid in a later published paper [20]. Results have shown that the changes of the physicochemical properties are more important than themselves when dealing with the deleterious nsSNP detection problem [20]. 

#### 4.1.4. Biochemical Properties

Recent studies [28–31] have shown that deleterious substitutions are likely to affect protein structure; therefore, a better understanding about the protein biochemical properties of protein structure changes may accelerate the detection of deleterious nsSNPs. SNAP computes a series of biochemical properties and uses them as important features to construct classification models [1]. The properties contain several binary features, such as whether there is an inflexible proline into an alpha-helix, and some continuous features, such as mass of wild-type and mutant residues.

### 4.2. Structure-Based Information Facilitates the Prediction of Deleterious nsSNPs

Given a protein sequence data, structure-based deleterious nsSNPs prediction methods find the best match against a protein structure database. Some structural features are extracted using the information surrounding the site of substitution instead of detailed information at the atomic level; therefore, if there is not a perfect match for a query protein in the protein structure database, the structure of a homologous protein can be used. 

Mapping of an nsSNP to a known 3D structure reveals whether the replacement is likely to destroy the hydrophobic property of a protein, electrostatic interactions, interactions with ligands, or other important features of a protein [2]. Structural features performed by PolyPhen are based on the use of several structural parameters suggested previously [32–34]. PolyPhen uses the Dictionary of Protein Secondary Structure (DSSP) database [35] to obtain some structural parameters for the mapped amino acid residues, such as secondary structure, solvent accessible surface area absolute value. The solvent accessible surface area (SASA) is the surface area of a molecule which is accessible to a solvent and used to improve prediction of protein secondary structure [36, 37]. PolyPhen-2 refines the structural parameters using a feature selection mechanism and chooses three important structural features among thirteen candidate structural features. The selected features are normalized accessible surface area of amino acid residue, crystallographic beta-factor reflecting conformational mobility of the wild-type amino acid residue, and change in accessible surface area propensity for buried residues [18]. 

Methods solely based on protein structure features provide fewer predictions than methods using sequence-based features because there are far fewer protein structures than sequences for which homology can be found [8]. It is reported that the ratio of methods using sequence-based features to all the existing methods is as high as 81%, while the ratio for methods using only structure-based features is only 14% [8]. Independently consideration of isolated protein structure sometimes may lead to misleading prediction, because the proteins often interacted with others. Thus, new methods tend to use sequence-based information as the main features and structure-based information as the supporting features to operate the deleterious nsSNP detection problem.

### 4.3. Annotations Can Enhance the Prediction Power for Identifying Deleterious nsSNPs

Annotations can be used as supplementary features to enhance the prediction power for identifying deleterious nsSNPs. The SwissProt database annotates the positions of a protein that are located in the active site, involved in ligand binding, part of a disulfide bridge, or involved in other protein-protein interactions. Annotations can enhance the prediction power when incorporating with other features, such as sequence-based predictions of secondary structure and solvent accessibility [38, 39]. PolyPhen adds the SwissProt feature table terms to the final prediction rules, and MutationTaster and SNAP also utilize the SwissProt annotations as features to predict the deleteriousness of the query nsSNPs. 

Besides annotations from published databases, the predicted deleterious score given by existing wide-accepted prediction tools can be treated as preliminary annotation for the prediction. For example, SNAP algorithm makes use of SIFT and PolyPhen prediction scores as classification features to enhance the prediction power. SNAP also determines whether the correct predictions made by their method overlapped with those covered by PolyPhen and SIFT [1]. 

## 5. Machine Learning Models for Classifying nsSNPs

Most predicting methods treat the identification of deleterious nsSNPs as a binary classification problem and adopt some popular binary classification machine learning algorithms, such as rule-based prediction model [2, 17], naïve Bayes classifier [5, 18], random forest [11], neural networks [1], and many others. After selecting suitable features, these prediction methods usually train and test on two types of datasets: a deleterious nsSNP set, which contains substitutions assumed to affect protein function, and a neutral set, which contains substitutions assumed to have no effect. During the training procedure, machine learning approaches are adopted to construct a classification and give a prediction score measuring the deleteriousness of an nsSNP. A prediction method should predict the substitutions in the deleterious nsSNP set to be damaging to protein function and predict the substitutions in the neutral set to be not related to protein function. Sometimes, a confident score is also provided to explain how confident the prediction result is. A bigger confident score means that the prediction is more approximate to the truth. Criteria to evaluate the prediction methods are mainly accuracy (ACC), false negative error rate (FN), and false positive rate (FP). False negative error rate is the percentage of nsSNP substitutions incorrectly predicted to be neutral, and false positive error is the percentage of neutral substitutions incorrectly predicted to affect protein function [8]. 

SIFT incorporates position-specific information by using sequence alignment and is intended specifically for predicting whether an amino acid substitution affects protein function. SIFT starts with a query protein sequence. Relying on the observation that proteins in the same subfamily have high conservation in conserved regions, SIFT selects sequences that are similar to the query sequence by adding the most similar sequence extracted from the PSI-BLAST results iteratively to the growing collection until conservation in the conserved regions decreases [17]. After the collection of similar sequences from multiple sequence alignment by PSI-BLAST, SIFT converts the alignment into a position-specific scoring matrix (PSSM) and calculates the probability of an amino acid appearing at a specified position. Using the position-specific probability estimation, SIFT assigns a decision rule to make the classification model. SIFT also provides a measure of confidence in the prediction. To assess confidence in the prediction, SIFT calculates a conservation value at each position in the alignment. PolyPhen also uses a rule-based decision mechanism to make the prediction for candidate nsSNPs. The rule is based on the analysis of the ability of various structural parameters and profile scores to discriminate between disease mutations and substitutions [2]. The rule-based prediction can be treated as prediction using decision trees, which belongs to the binary classification.

Quite different from PolyPhen, PolyPhen-2 adopts a naïve Bayes approach coupled with entropy-based discretization. The naïve Bayes approach can work as well as some machine learning approaches and contains only one parameter, which is Laplace estimators used for representing factored probabilities and smoothing [18]. MutationTaster also uses a naïve Bayes classifier, which predicts the potential candidate disease-associated nsSNPs. Different from other algorithms, MutationTaster chooses between three different prediction models, which are either aimed at “silent” synonymous or intronic alterations, at alterations affecting a single amino acid, or at alterations causing complex changes in the amino acid sequence.

MSRV provides a more realistic solution for identifying disease-associated nsSNPs. MSRV prioritizes mutations occurring in genetic regions to find those that are most likely to cause diseases. MSRV first partitions the training set to 20 subsets according to different type of amino acid and utilizes a sequential forward feature selection method to choose the valuable features for each subset among extracted 26 physiochemical and conservation features. Then, MSRV trains a decision tree for each subset and takes advantage of random forest algorithm for the multiple selection strategy. 

SNAP could potentially classify all nsSNPs in all proteins into deleterious (effect on function) and neutral (no effect) using sequence-based computationally acquired information alone. For each instance SNAP provides a reliability index, which is a well-calibrated measure reflecting the level of confidence of a particular prediction. SNAP uses an approximation of the rule of thumb for feature selection, and a standard feed-forward neural network with momentum term to build a classification model. SNAP also applies support vector machines (SVMs) for the prediction problem but receives a worse performance than a comparable neural network-based method.

## 6. Beyond Classification of nsSNPs

### 6.1. From Coding Mutations to Noncoding Mutations

Methods for predict deleteriousness of nsSNPs mainly focus on protein coding regions and conveniently use the properties derived from protein sequence or structure as classification features. Although nsSNPs in protein coding regions are important for studying the potential causative relationship between genetic variants and human inherited diseases, variants in intergenic regions, promoter regions, and intron regions can also strongly influence the phenotypic outcome [21]. In a recently published paper, Kjong-Van and Ting construct a new model named SInBaD (sequence-information-based decision-model) to evaluate any annotated human variant in all known exons, introns, splice junctions, and promoter regions. SInBaD uses nucleotide sequence conservation across multiple vertebrate species as features to find functional variants in regions other than just the coding regions. SinBaD builds three separate mathematical models for promoters, exons, and introns, using the human disease mutations annotated in human gene mutation database as the training dataset for functional variants. The authors perform deleterious variant analysis on four of the currently available individual human genomes and find out that there is considerable amount of predicted deleterious variants in promoter and intron region, especially the number of predicted deleterious variants in promoter region is almost 40% of the number of predicted deleterious variants in all regions. 

Besides SinBaD, GERP also tries to overcome the limitation for noncoding mutation prediction. GERP identifies constrained elements in multiple alignments by quantifying substitution deficits, which represent that substitutions may occur if the element is neutral DNA and do not occur if the element is under functional constraint. These deficits, referred to as “Rejected Substitutions,” are a natural measure of constraint that reflects the strength of past purifying selection on the element [24]. Although GERP is an algorithm to infer the constrained region, it gives each location a substitution rejection score which can be further used as a conservation score for identifying deleterious variants.

### 6.2. From Deleterious Classification to Disease-Specific Prioritization

The nsSNP deleterious prediction becomes more and more wholesome when using valuable feature information, sequence information, and annotations from known database. Though effective, all these methods formulate the identification of nsSNPs that are associated with diseases as a classification problem and give no information about what specific disease the nsSNP is associated with. Therefore, the classification results of these methods can only provide limited information to practical applications. For example, an nsSNP *i* with the highest deleterious prediction score may totally change the function of the corresponding protein and have a strong relation with disease *A*. However, it is impossible that the nsSNP *i* is strongly related to all the other diseases. Therefore, disease-specific prediction models are constructed according to the features of variants, information of diseases, known disease-related variants, or other available disease-specific information. Wu et al. use ensemble learning methods to construct a prediction mechanism for disease-specific nsSNPs identification [40] and demonstrate high accuracy of their method. A biological knowledge-based mining platform for genomic and genetic studies using sequence data (KGGSeq) is also a disease-specific prioritization method which makes effort to find the causal mutations for a particular Mendelian disease among millions of variants.

### 6.3. From Common Variant Detection to Rare Variant Detection

Recently, the popular common-disease common-variant (CDCV) hypothesis that assumes the etiology of common diseases is intervened by commonly occurring genetic variants with small to modest effects has been challenged by the fact that both common variants and rare mutations may be involved in the pathogenesis of common diseases. In fact, studies have already revealed that the presence of multiple rare variants may augment the risk of some diseases. Corresponding to these findings, a common-disease rare-variant (CDVR) hypothesis that indicates that multiple rare variants can also serve as the main factor to influence some common diseases has been proposed. Therefore, deleterious rare variant prediction becomes a new challenge. 

KGGSeq is modeled to a comprehensive three-level framework to combine a number of filtrations and prioritization functions into one analysis procedure for exome sequencing-based discovery of human Mendelian disease genes. The framework is composed by several rules to filter and prioritize variants at three different levels: genetic level, variant-gene level, and knowledge level. KGGSeq can implement rare variant detection for Mendelian disease. During a rare variant detection, KGGSeq uses some genetic information and rules to filter the candidate variants, as well as a mechanism to delete common variants deposited in public databases (including the 1000 Genomes Project and NCBI dbSNP) as well as existing in the in-house datasets according to an adjustable allele frequency threshold. KGGSeq also incorporates PPI, pathway, and literature information to narrow down the candidate rare variants.

### 6.4. From Single Prediction Score to Integration of Multiple Prediction Scores

More and more deleterious variant prediction methods are developed using different types of features and different training set. As each method has its own strength and weakness, it has been suggested that the combination of some of the prediction scores may enhance the accuracy for predicting a variant. Database for nonsynonymous SNPs' functional predictions (dbNSFP) follows this idea to first build an integrated database of functional predictions from multiple algorithms for the comprehensive collection of human nsSNPs. KGGSeq adopts the prediction scores from four popular algorithms (SIFT, PolyPhen-2, LRT, and MutationTaster) along with a conservation score (PhyloP) published by dbNSFP. KGGSeq uses these five scores as prediction features to train a logistic regression model and find these scores are in weak or moderate correlation. When individually operating the algorithm, MutationTaster outperforms than the other four prediction algorithms. In addition, the combination of predictions by all the five deleterious scores can provide better performance than individual scores as well as combined prediction by part of the deleteriousness scores.

## 7. Conclusions and Discussion

Deleterious variants detection becomes a more and more popular issue for research and guiding real experiment. In this paper we summarize the database for collecting nsSNP data, existing deleterious nsSNPs prediction methods, prediction features conducted in prediction model, and prediction algorithms to distinguish the deleterious nsSNPs. We discuss computational methods that use comparative genomics to predict deleteriousness in both coding and noncoding DNA, methods for disease-specific nsSNP detection, and methods for rare variant detection. We suggest using multiple prediction algorithms, as well as more available molecule level information may help to enhance the prediction power.

Although the prediction of deleterious nsSNPs seems to be more and more accurate when integrating more valuable information of nsSNPs, there still exist some challenges to deal with. Conservation scores are used by most of the prediction methods as main features to predict the functional effects of a candidate variant. However, not all the deleterious variants are in the constraint region or conserved among multiple sequence alignment. As a result, the nonconserved variants are difficult to identify using existing methods. Accuracy assessment is another problem. During the prediction, deleterious variants and neutral variants are collected from published database, such as OMIM and SWISSPROT. However, whether the so-called deleterious variants are really deleterious or not and whether the so-called neutral variants are really neutral or not may strongly affect the construction of predict model and the final accuracy measurement of the model. In addition, even if a variant is predicted to be deleterious with a strong confidence, the information about which disease the variant is related to and which disease the variant has a casual relation with is still missing. Facts show that variants in noncoding region can strongly influence the phenotypic outcome, and more algorithms for noncoding region deleterious variant detection using more available features besides conservation scores should be designed for further studying the casual relationship between noncoding variants and diseases. Furthermore, standard evaluation rules should be proposed for better comparing the existing deleterious variant prediction methods. 

As a suggestion, more molecule level protein information or gene information should be merged with existing features to further strengthen the prediction power. As the protein products of genes responsible for the same or phenotypically similar disorders tend to physically interact with each other so as to carry out certain biological functions [23], PPI data could be considered. Genes sharing similar GO terms trend to have similar functions; thus, gene-gene similarity calculated using GO terms could provide another choice. Moreover, pathway information can be included based on the fact that causative genes of the same (or phenotypically similar) diseases are inclined to distribute within the same pathways.

## Figures and Tables

**Table 1:** Database for collecting nsSNP data.

Database	Website	Reference ID
Online Mendelian Inheritance in Man (OMIM)	http://www.omim.org/	[22]
Human Gene Mutation Database (HGMD)	http://www.hgmd.cf.ac.uk/ac/index.php	[12]
UniPROT/SWISS-PROT database	http://www.uniprot.org/	[13]
Human Genome Variation database (HGVbase)	http://hgvbase.cgb.ki.se	[14]
Single-nucleotide polymorphism database (dbSNP)	http://www.ncbi.nlm.nih.gov/snp	[15]
Protein Mutant Database (PMD)	http://pmd.ddbj.nig.ac.jp	[16]
Database for nonsynonymous SNPs' functional predictions (dbNSFP)	http://sites.google.com/site/jpopgen/dbNSFP	[9]

**Table 2:** Tools for deleterious variant detection.

Method	Website	Features	Method description	Reference ID
SIFT	http://sift.bii.a-star.edu.sg/	Sequence based	Statistical method using PSSM with Dirichlet priors	[17]
PolyPhen	http://genetics.bwh.harvard.edu/pph/index.html	Sequence based, structure based, annotation	Rule-based model	[2]
SNAP	http://www.rostlab.org/services/SNAP/	Sequence based, annotation	Standard feed-forward neural networks with momentum term	[1]
MSRV	http://bioinfo.au.tsinghua.edu.cn/member/ruijiang/english/software.html	Sequence based	Multiple selection rule voting strategy using random forest	[11]
LRT	http://www.genetics.wustl.edu/jflab/lrt_query.html	Sequence based	Log ratio test	[19]
PolyPhen-2	http://genetics.bwh.harvard.edu/pph2/index.shtml	Sequence based, structure based	Naïve Bayes approach coupled with entropy-based discretization	[18]
MutationTaster	http://www.mutationtaster.org/	Sequence based, annotation	Naïve bayes model based on integrated data source	[5]
KGGSeq	http://statgenpro.psychiatry.hku.hk/limx/kggseq/	Sequence based, annotation	A three-level framework to combine a number of filtration and prioritization functions	[23]
SInBaD	http://tingchenlab.cmb.usc.edu/sinbad/	Sequence based	Separate mathematical models for promoters, exons, and introns, using logistic regression algorithm	[21]
GERP (score)	http://mendel.stanford.edu/sidowlab/downloads/gerp/index.html	Sequence based	A “Rejected Substitutions” score computation to infer the constrained region	[24]
PhyloP (score)	http://hgdownload.cse.ucsc.edu/goldenPath/hg18/phyloP44way	Sequence based	An exact *P* value computation under a continuous Markov substitution model	[10]
